# Evaluating the In Vivo Virulence of Environmental *Pseudomonas aeruginosa* Using Microinjection Model of Zebrafish (*Danio rerio*)

**DOI:** 10.3390/antibiotics12121740

**Published:** 2023-12-15

**Authors:** Edit Kaszab, Dongze Jiang, István Szabó, Balázs Kriszt, Béla Urbányi, Sándor Szoboszlay, Rózsa Sebők, Illés Bock, Zsolt Csenki-Bakos

**Affiliations:** 1 Department of Environmental Safety, Institute of Aquaculture and Environmental Safety, Hungarian University of Agriculture and Life Sciences, 2100 Gödöllő, Hungary; kaszab.edit@uni-mate.hu (E.K.); jiang.dongze@phd.uni-mate.hu (D.J.); szoboszlay.sandor@uni-mate.hu (S.S.); sebok.rozsa.eszter@phd.uni-mate.hu (R.S.); 2 Department of Environmental Toxicology, Institute of Aquaculture and Environmental Safety, Hungarian University of Agriculture and Life Sciences, 2100 Gödöllő, Hungary; szabo.istvan.temi@uni-mate.hu (I.S.); bock.illes@uni-mate.hu (I.B.); csenki-bakos.zsolt.imre@uni-mate.hu (Z.C.-B.); 3 Department of Aquaculture, Institute of Aquaculture and Environmental Safety, Hungarian University of Agriculture and Life Sciences, 2100 Gödöllő, Hungary; urbanyi.bela@uni-mate.hu

**Keywords:** *Danio rerio*, *Pseudomonas aeruginosa*, microinjection, infection model, antibiotic resistance, virulence

## Abstract

(1) Background: Microinjection of zebrafish (*Danio rerio*) embryos offers a promising model for studying the virulence and potential environmental risks associated with *Pseudomonas aeruginosa*. (2) Methods: This work aimed to develop a *P. aeruginosa* infection model using two parallel exposition pathways on zebrafish larvae with microinjection into the yolk and the perivitelline space to simultaneously detect the invasive and cytotoxic features of the examined strains. The microinjection infection model was validated with 15 environmental and clinical strains of *P. aeruginosa* of various origins, antibiotic resistance profiles, genotypes and phenotypes: both exposition pathways were optimized with a series of bacterial dilutions, different drop sizes (injection volumes) and incubation periods. Besides mortality, sublethal symptoms of the treated embryos were detected and analyzed. (3) Results: According to the statistical evaluation of our results, the optimal parameters (dilution, drop size and incubation period) were determined. (4) Conclusions: The tested zebrafish embryo microinjection infection model is now ready for use to determine the in vivo virulence and ecological risk of environmental *P. aeruginosa*.

## 1. Introduction

*Pseudomonas aeruginosa* is one of the most widely investigated model organisms to evaluate the specific interactions between the microbe and the host [1]. In clinical environments, *P. aeruginosa* is known as a highly virulent opportunistic microorganism responsible for severe nosocomial infections with increased mortality compared to other Gram-negative bacteria [2] as it was verified in a 13-year (2002–2015) prospective cohort study on bloodstream infections in the USA [3]. At the same time, it can survive in diverse ecological niches including soil, surface water and groundwater [4,5] and plant and animal tissues [6], and under special circumstances, it can become the dominant member of the microbial community [7]. Several molecular and phenotypic traits and transcriptional factors (TFs) are linked to the virulence of *P. aeruginosa* [8] including cell-associated and extracellular virulence factors [9] and exotoxin/exoenzyme production [10] such as the *oprL* and *toxA* [11]. Many of them are encoded within the core genome [12,13] (among genes that are essential across all strains of a given species under all relevant growing conditions) [13], but the variation among isolates can be partly linked to their population structure [14]. Epidemiological typing methods, such as multilocus-sequence typing (MLST), often show a good correlation with virulence profiles [15]. At the same time, no correlation was found between the genetic population structure and the phenotypic expression of antibiotic resistance [16]. Despite the well-known ecological role of *P. aeruginosa* as a major opportunistic fish pathogen [17] of various species such as *Oreochromis niloticus* and *Clarias gariepinus* [11], environmental strains are often considered as “non-pathogenic” based on the lack of several virulence-related genes [18] or their non-hemolytic phenotype [19], although no apathogenic variants of the species have been reported so far [20]. As a consequence, environmental strains are routinely used in environmental biotechnology [21,22]. To clarify the human health and ecological concerns of environmental *P. aeruginosa* strains, rapid and reliable in vivo virulence models are still playing a critical role. 

Zebrafish (*Danio rerio*) embryos have recently become one of the most important vertebrate animal models for the study of host–pathogen interactions and infectious diseases [23]. The virulence and infection mechanisms of several viral, fungal and bacterial species, including *P. aeruginosa* [24,25], were studied in zebrafish, taking advantage of the optical transparency of the embryos [26] and the parallels between zebrafish and human innate immune cells [27]. In these in vivo models, infections occur artificially—microorganisms are introduced into the embryos by microinjection.

In most cases, microinjections can be made into the caudal vein, the Duct of Cuvier, the hindbrain ventricle, the tail muscle, the notochord or the otic vesicle of the 1–3 dpf embryos [28] or, in the case of slow-growing bacteria, into the yolk of 1–1024 cell stage embryos [29]. The advantage of the microinjection method is that both systemic and localized infections can be examined depending on the site chosen for infection [23]. Moreover, zebrafish microinjection enables the detection of sublethal symptoms besides mortality [30], giving a more comprehensive insight into host–pathogen interactions. However, with the exception of the injection into the early embryo stage, the execution of these methods requires a lot of practice and can be considered slow [31]. Another disadvantage of embryo microinjection is the direct introduction of microorganisms, which determines whether the infection can become systematic and induce lethality or remain localized [25]. As an alternative, bath immersion [25,32,33] was developed as an easy-to-handle method that simulates a natural infection route and was further developed into a wound infection model with the usage of tail-injured embryos [31,34]. Despite the increasing number of virulence-testing methods using different routes, none of the above-mentioned techniques can provide simultaneous information about the invasive and cytotoxic features of a given strain. To get a reliable overview of the complex virulence performance of *P. aeruginosa*, a more sophisticated in vivo method is required that enables the determination of the infectivity of a given strain and the prediction of its environmental health risks.

In this work, a new testing and evaluation method is advanced and demonstrated, which combines different zebrafish embryo microinjection methods to evaluate the virulence of *P. aeruginosa*. The developed methodology can be suitable both for determining the infectivity of fast-growing microorganisms and for testing the adverse effects that develop after early infection. The method was optimized and tested using *P. aeruginosa* strains isolated from various environmental niches and to a lesser extent with clinical reference strains.

## 2. Results

### 2.1. Method Development

The first step of the method development was the optimization of the minimum observation period and the bacterial cell count (calculated from the bacterial density and the drop size) used for injection. At this stage, two environmental *P. aeruginosa* were chosen for testing: both of them harbored a set of virulence determinants, but one of them was previously determined as virulent (strain P66) and the other as avirulent (strain P14) based on their in vivo virulence on *G. mellonella* [35]. According to their antibiotic susceptibility profile, P66 had a sensitive phenotype, while P14 had a resistant phenotype [35].

To determine the early-stage symptoms, the yolk-injection method was performed immediately after fertilization. This rapid infection protocol enables the injection of a large number of eggs in a short period of time and can be easily implemented without a holder [30]. Zebrafish was described as a relatively resistant species to Pseudomonads, and therefore, in previous studies, a large inoculum (above 1000 bacteria per embryo) was used to induce host killing [25]. At the same time, yolk injection is specifically recommended for testing slow-growing microorganisms [28]. Therefore, during the preliminary phase, the bacterial density of the stock suspension of fast-growing *P. aeruginosa* was set to OD600 = 0.6 ± 0.02 (equivalent to 4.8 × 10^8^ ± 3.33% CFU/mL (as it was verified with enumeration on Luria-Bertani (LB) agar) and was serially diluted to reach the minimum infective dose (MID) (10^2^ CFU) [36,37] in the final injection volume. Moreover, additional levels of dilutions (10^−2^–10^−4^) were set to determine sublethal effects and to evaluate environmentally relevant *P. aeruginosa* cell counts which are commonly detectable in various environmental niches such as soil [38], compost [39], surface water [40] and groundwater [41].

To evaluate the invasive features of the examined strains, microinjection was performed directly into PV (perivitelline) as well. Since the chorion pore size of zebrafish (0.77 µm) [42] is smaller than the average cell size of *P. aeruginosa* (0.5 to 1.0 µm by 1.0 to 5.0 µm) [43], the micro-environment of the infection model is theoretically sealed and prevents the cross infection between embryos until they are hatched (around 72 hpf) which can be further improved if we use one well to cultivate per embryo. For the PV infection route, three different levels of bacterial dilutions were used representing MID (minimum infectious dose that is approximately 10 bacterial cells) and additionally one lower (10^−1^) and one higher (10^−3^) levels of dilutions. The applied levels of bacterial dilutions and their relevant cell counts are summarized in Table 1.

The drop size used for microinjection is critical in the posttreatment survival of zebrafish larvae and simultaneously influences the infectious dose of the tested bacterial strain. In the case of fish embryos, the maximum volume for injection that does not significantly influence the survival of the larvae is 10% of the total yolk volume [44,45], which is approximately 4.2 nL (equivalent to a 200 µm diameter droplet) for zebrafish larvae [46]. This test volume was comprehensively tested in previous experiments with no significant mortality or developmental disorders [30], and therefore, it is considered to be safe for application. To avoid damage to the test organism, besides this maximum volume, two smaller, easily adjustable drop sizes, 100 µm in diameter (0.52 nL) and 150 µm in diameter (1.77 nL), were chosen for testing.

Altogether, to ensure the comprehensive analysis, the examined environmental strains (P66 and P14) and the non-inoculated control medium were tested using two infection routes (PV, Y), three chosen drop sizes and all relevant concentrations of bacterial suspensions, meaning 27 different settings. The sublethal symptoms of the embryos were registered every 24 h until they were hatched (after 24/48/72 h incubation); therefore, altogether 81 endpoints were determined. *P. aeruginosa* infection of the perished embryos was verified with the re-isolation of the infectious strains as it was recommended [47].

**Figure 1:**
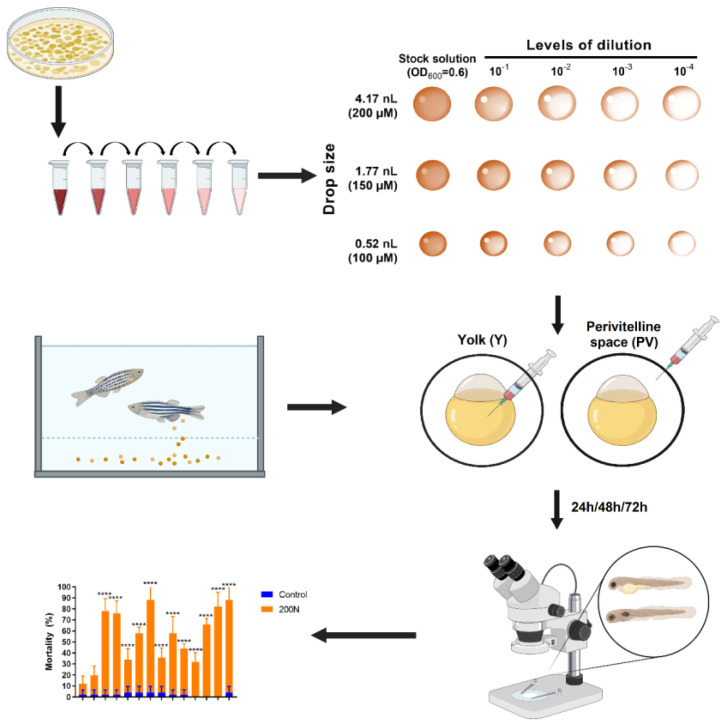
The flowchart of the microinjection protocol used for preliminary testing with the relevant cell counts (doses). GP: <0.0001 (****).

The flowchart of the microinjection protocol applied for preliminary testing is summarized in Figure 1.

### 2.2. Optimization of the Combined Zebrafish Microinjection Virulence Model

An optimal animal model used for virulence testing can be characterized by having a low cost, high infection success, ease of infection initiation, a low mortality rate of the test organism and a reasonable expense [48]. To meet these requirements, the incubation time, bacterial concentrations and droplet size of the newly developed protocol had to be optimized to reach a high infection success with relatively low mortality in a short period of time with easy implementation and, at the same time, to have a clear, statistically significant difference compared to the untreated control group. To reach these goals, the mean mortality values (%) of all different settings (incubation time, levels of dilutions, drop size) of zebrafish embryo groups treated with PV or Y injection of P14 and P66 *P. aeruginosa* strains were statistically evaluated.

#### 2.2.1. Determination of the Optimal Incubation Time

**Figure 2:**
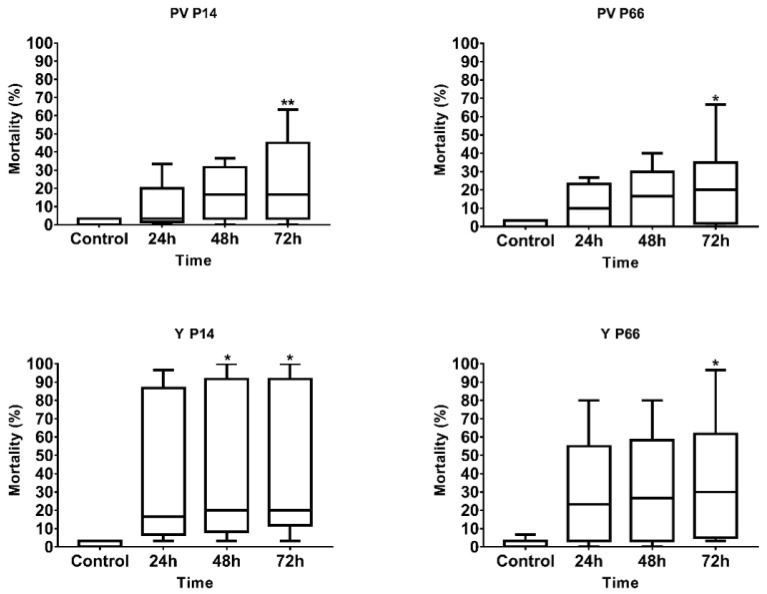
Mortality results of the examined *P. aeruginosa* strains during optimization of zebrafish microinjection model with different times of expositions (24, 48, 72 h). Groups of 10, 3 replicates. Mean values were analyzed with one-way ANOVA followed by Dunnett’s multiple-comparison test at 95% confidence interval; GP: 0.1234 (ns), 0.0332 (*), 0.0021 (**), PV—perivitelline injection; Y—yolk.

According to the multiple comparisons of the mean mortality values at 24 h, 48 h and 72 h of PV/N groups and the control group (summarized in Figure 2), a statistically significant difference (GP: 0.0332–0.0021) occurred after 72 h of incubation irrespective of the drop size and the dilution used for treatment. Therefore, the first step of optimization supported the 72 h-long incubation of zebrafish larvae, which is equivalent to the hatching period. In further correlation analysis, the mortality results with shorter (24 h and 48 h) incubation were excluded to decrease the standard deviation of data.

#### 2.2.2. Determination of the Optimal Level of Dilution of *P. aeruginosa*

**Figure 3:**
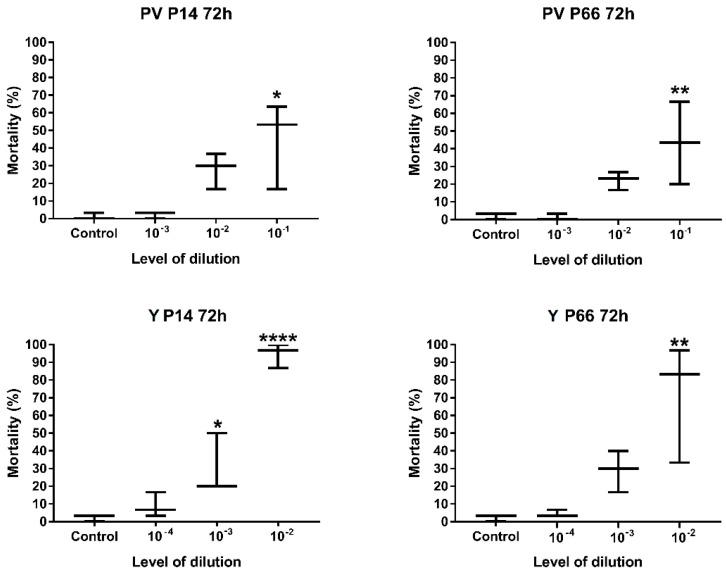
Mortality results of the examined *P. aeruginosa* strains during optimization of zebrafish microinjection model with different levels dilutions of bacterial strains (10^−1^/10^−2^–10^−3^/10^−4^). Groups of 10, 3 replicates. Mean values were analyzed with one-way ANOVA followed by Dunnett’s multiple-comparison test at 95% confidence interval; GP: 0.1234 (ns), 0.0332 (*), 0.0021 (**), 0.0001 (****). PV—perivitelline injection; Y—yolk injection.

To determine the optimal level of bacterial dilution, results of 72 h-long incubation with different (10^−1^–10^−4^) dilution levels were compared to the mortality of the control group. According to the statistical analysis using one-way ANOVA with Dunnett’s multiple-comparison tests, only the highest examined bacterial concentrations (i.e., the lowest levels of dilutions) caused a significantly different level of mortality compared to the non-treated control (Figure 3). Therefore, the recommended levels of tenfold dilutions, using a freshly prepared stock solution of *P. aeruginosa* with OD600 = 0.6 ± 0.02 density, are 10^−1^ (PV) and 10^−2^ (Y).

#### 2.2.3. Determination of the Optimal Drop Size

**Figure 4:**
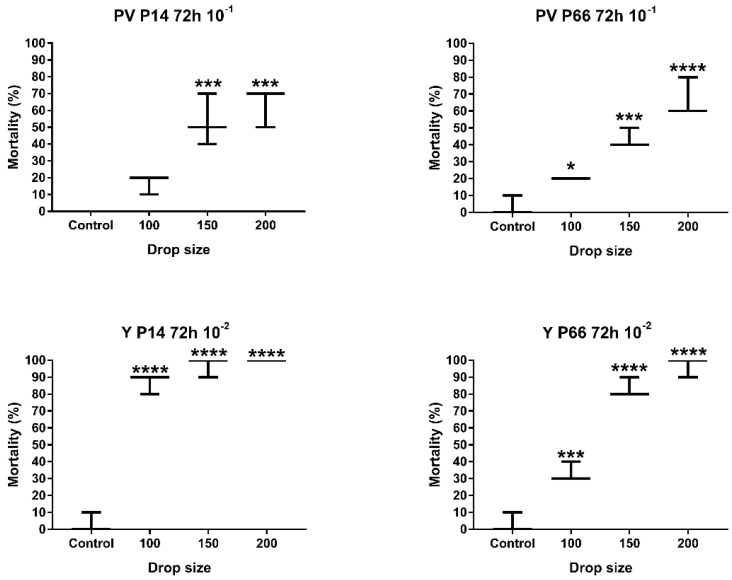
Mortality results of the examined *P. aeruginosa* strains during optimization of zebrafish microinjection model with different drop sizes (100, 150, 200 µL). Groups of 10, 3 replicates. Mean values were analyzed with one-way ANOVA followed by Dunnett’s multiple-comparison test at 95% confidence interval; GP: 0.1234 (ns), 0.0332 (*), 0.0002 (***), 0.0001 (****). PV—perivitelline injection; Y—yolk injection.

The determination of the optimal drop size of the infectious material used for microinjection was calculated using the results of the previously determined 72 h incubation and 10^−1^ (PV) and 10^−2^ (Y) levels of dilution. Based on our results, only the smallest drop size (100 µM (0.52 nL)) had a statistically insignificant effect compared to the control group, while 150 µM (1.77 nL) and 200 µM (4.17 nL) drop sizes had significant (GP: 0.0002–0.0001) effects on the mortality of the zebrafish embryos (Figure 4). Based on the general requirements for virulence testing, using only one drop size would increase the speed of the process and decrease the occurrence of errors. Considering that the larger drop size increases the chance of causing a lesion in the embryo during microinjection, our decision was to apply the smaller, but still significantly effective, 150 µM drop size for validation.

#### 2.2.4. Development of the Recommended Infection Model

Based on the optimization process and the statistical analysis, the recommended method for the combined microinjection protocol of zebrafish larvae with environmental *P. aeruginosa* using parallel perivitelline (PV) and yolk (Y) exposition is as follows: two different levels of dilutions of the overnight OD600 = 0.6 bacterial stock solution (10^−1^ for Y and 10^−2^ for PV) in 150 µL drop size and 72 h of incubation.

### 2.3. Validation of the Developed Microinjection Method with a Set of P. aeruginosa Isolates

**Figure 5:**
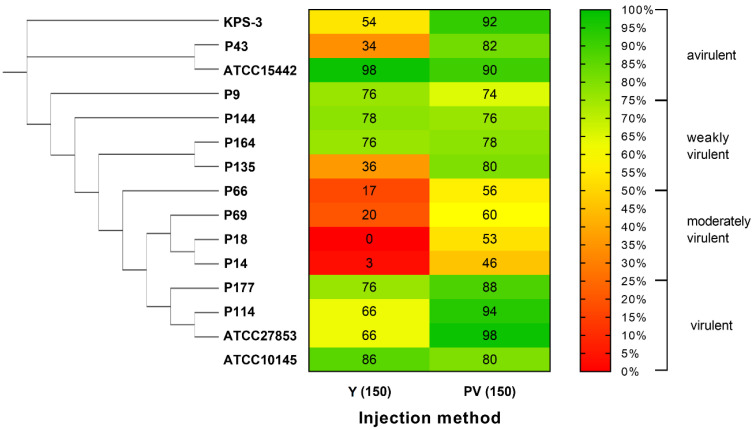
Virulence of the examined environmental and clinical *P. aeruginosa* strains using the combined microinjection virulence model and the spread of the in vivo virulence features across the phylogenetic tree generated by the multilocus sequence types (STs) of the examined strains using PUBMLST, the public database for molecular typing and microbial genome diversity (ATCC10145 was not classified). Y—yolk injection; PV—perivitelline injection. Avirulent: survival rate of 75–100%; weakly virulent: survival rate of 50–74%; moderately virulent: survival rate of 25–49%; virulent: survival rate of 0–24%.

The newly developed infection protocol was validated using 15 environmental and clinical strains of *P. aeruginosa* representing various phenotypic, genetic and phylogenetic traits. Similar to the previously performed *G. mellonella* virulence assay of environmental *P. aeruginosa* [33], a categorization of four groups was used for the evaluation after 72 h of incubation: avirulent (with a survival rate of 75–100%), weakly virulent (survival rate: 50–74%), moderately virulent (survival rate: 25–49%) and virulent (survival rate: 0–24%). These categories were set according to the evaluation methods of the in vivo virulence test using *G. mellonella* [49]. The virulence categories of the examined strains treated with Y and PV exposition are summarized in Figure 5. According to our results, yolk injection induced a significantly higher rate of mortality as the infectious strain was directly introduced to the yolk, while perivitelline infection showed the ability of the bacterial strain to actively invade the host. Pronounced differences in both exposition routes were detected across the phylogenetic tree supporting the theory that several virulence traits can be linked to population structure [14]. At the same time, no correlation was found between the phenotypic antibiotic resistance profiles and the in vivo virulence of the examined strains. Based on the parallel analysis of two different types of exposition, the environmental and ecological hazard of a given *P. aeruginosa* isolate is suggested to be considered as “high” if the categorization of virulence reaches at least a moderate level (with a survival rate of 50% or lower) in both types of microinjection routes and “moderate” if the survival rate reaches 25% in both exposition pathways.

### 2.4. Sublethal Symptoms Detected at the End of Incubation

In addition to mortality, phenotypic malformations were also observed in surviving embryos. In general, despite the fact that all embryos were treated, the developed symptoms were not uniform within the treatment groups, and there were individuals in every case that did not show any phenotypic differences compared to the control. Based on these findings, phenotypic malformations were not taken into account in the final process of virulence evaluation.

**Figure 6:**
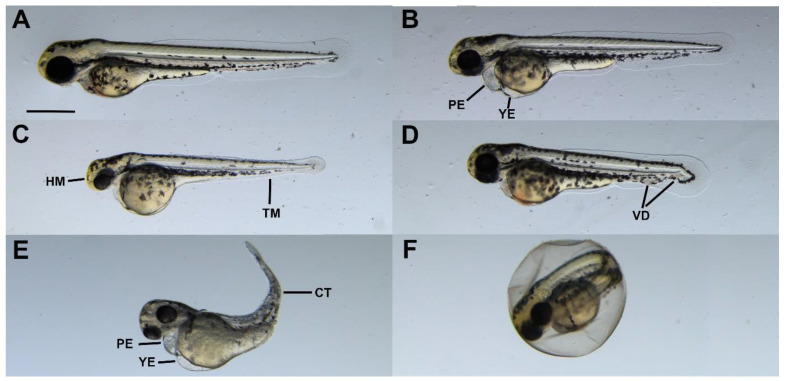
Representative phenotypic malformations caused by *Pseudomonas aeruginosa* in 72 hpf zebrafish embryos. (**A**) Control, (**B**) pericardial and yolk edema (injected strain: P14), (**C**) head and tail malformations (injected strain: P132), (**D**) vascular disorders in the tail region (injected strain: P43), (**E**) edemas and curved tail (injected strain: P26), (**F**) hatching disorders (injected strain: P66). Abbreviations: PE: pericardial edema; YE: yolk edema; HM: head malformation; TM: tail malformation; VD: vascular disorder; CT: curved tail. Scale bar: 500 µM.

The observed malformations (Figure 6) were very similar to the symptoms described in the literature: yolk and pericardial edema were the most characteristic symptoms [33,50,51]. In addition to these, the frequently observed morphological abnormalities included small and not well-defined head and tail regions, bent bodies and malformed blood vessels in the tail region. Some of the embryos could not hatch from the egg.

### 2.5. Methodological Summary of the Newly Developed, Combined Virulence Model

**Figure 7:**
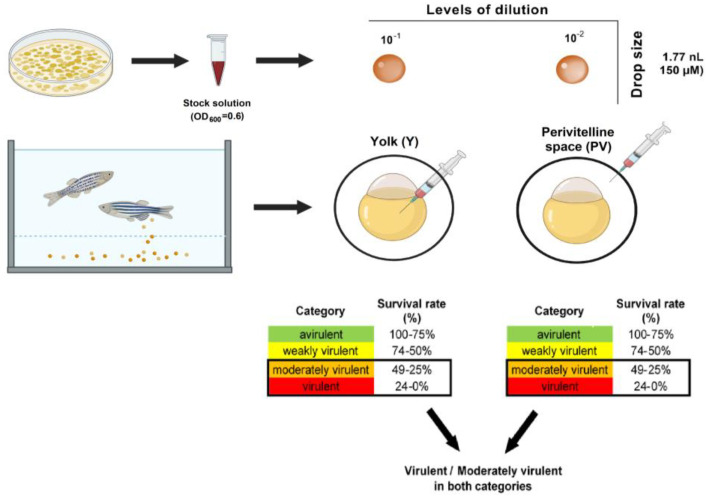
The recommended flowchart of the newly developed virulence infection model using zebrafish larvae to evaluate the virulence of *P. aeruginosa*.

Based on the comprehensive analysis of the combined zebrafish injection model using 15 representatives of species *P. aeruginosa*, the optimized and validated methodology was set. The flowchart of the recommended infection protocol is summarized in Figure 7.

## 3. Discussion

In the last decades, several virulence models were developed and validated for in vivo *P. aeruginosa* virulence testing [52]. As species *P. aeruginosa* uses a variety of virulence determinants to combat host defense, pathogenesis was evaluated using several, evolutionary divergent host species such as rodents [53,54,55], *Drosophila melanogaster* [56], *Caenorhabditis elegans* [57,58], *Galleria mellonella* [49,59] and zebrafish (*Danio rerio*) [25,31,50,60,61]. These test organisms such as *D. rerio* were chosen for virulence assays because they share physiological and genetic similarities with humans, and therefore, they can support medical advancements in human therapy [62]. Compared to other vertebrate models, a major advantage of zebrafish larvae testing is that, according to European legislation, before the independent feeding stage of the larvae, zebrafish larval models are not regulated as experiments with laboratory animals [63,64]. Therefore, zebrafish models using <120 hpf embryos have low ethical considerations [65]. Moreover, similar to *C. elegans* and *D. melanogaster*, zebrafish larvae microinjection tests have a short timeframe, low maintenance cost and high throughput [52] which are major advantages in virulence testing [48].

Compared to the above-mentioned *P. aeruginosa* virulence assays, the newly developed protocol has further advantages: it can be performed within 72 h, and it requires only two levels of bacterial dilutions and an extremely small volume of the tested bacterial strain. Using two parallel exposition pathways to test both invasive and cytotoxic features, and with the possibility to analyze sublethal symptoms, the newly developed protocol enables a complex evaluation of in vivo virulence of *P. aeruginosa*.

During preliminary testing, two environmental *P. aeruginosa* strains with different phenotypic traits and antibiotic susceptibility profiles were used that were previously determined as virulent (P66—95% mortality) and avirulent (P14—5% mortality) on *G. mellonella* (Table 2) [35]. Based on our results using zebrafish microinjection, the examined two strains were not statistically different in their virulence on zebrafish larvae: both isolates were moderately virulent in PV and highly virulent in Y injection. During the extensive testing of 15 environmental and clinical *P. aeruginosa* strains, similar results were obtained: compared to PV injection, Y microinjection induced a significantly higher rate of mortality in 9 out of 15 cases, even though the bacterial cell count of Y injection was lower. This higher level of mortality can be explained by the direct introduction to the yolk.

Compared to the previously performed *G. mellonella* virulence test using the same *P. aeruginosa* strains, major differences were revealed in mortality in both PV and Y exposition (see Supplementary Table S1). In the scientific literature, there is only one available study about the comparison of zebrafish larvae microinjection and *G. mellonella* virulence testing on *K. pneumoniae* that showed host-dependent differences in virulence, explained by the different infection dynamics of the examined strains [66]. Our study follows this previous finding and underlines that there can be major differences between virulence results on different host organisms due to the presence/absence of host-specific virulence factors [50].

MLST analysis of the examined *P. aeruginosa* strains revealed that clinical and environmental *P. aeruginosa* strains that belong to closely related sequence types (STs) tend to have similar virulence patterns on zebrafish larvae and *G. mellonella* irrespective of their antibiotic susceptibility (Figure 5 and Supplementary Table S1). This finding further verifies that the population structure of *P. aeruginosa* may also play a role in the virulence performance of a given strain [14].

The virulence of environmental strains of *P. aeruginosa* is commonly determined by molecular techniques aiming to detect a set of virulence determinants [12,15,39,67]; in vivo assays can be rarely found [68,69]. The zebrafish microinjection model presented in our study has the advantage of being tested and validated with comprehensively characterized *P. aeruginosa* strains not only from strain collections and clinical settings but of environmental origin, too. Therefore, future application is possible not only for clinical but also for environmental strains. However, there are limitations to our method as it was optimized for a fast-growing Gram-negative bacterium (*P. aeruginosa*) only, with a limited number of clinical strains. Further validation may be required to evaluate if the protocol is applicable for testing clinical strains, other (slow-growing) Gram-negative, Gram-positive bacterial species or fungi.

## 4. Materials and Methods

### 4.1. Ethics Statement

The Animal Protocol (2013) was approved under the Hungarian Government Regulation on animal experiments (40/2013 (II.4)), and all studies were completed before the treated individuals would have reached the free-feeding stage.

### 4.2. Bacterial Strains

For the validation of the newly developed virulence model, a set of 15 *P. aeruginosa* isolates were chosen, representing a variety of origin, phenotypic and genetic traits, multilocus sequence types (MLSTs), antibiotic resistance, virulence factors, biofilm-forming ability and motility and in vivo virulence on *Galleria mellonella* (wax moth) that was determined and published previously [35] (Table 1). ATCC15442 was used as a negative control as this environmental strain was stated to be non-pathogenic [70]. Environmental and clinical *P. aeruginosa* strains were obtained from the National Collection of Agricultural and Industrial Microorganisms (NCAIM), Hungary, and from the collection of the Hungarian University of Agriculture and Life Sciences (MATE), Department of Environmental Safety. All examined *P. aeruginosa* strains were identified at species level with the PCR targeting the variable regions V2 and V8 of 16S rDNA, as it was described previously [71]. For preliminary experiments, aiming at the optimization of the microinjection method, two environmental strains (P14 and P66 highlighted in Table 2) were chosen to represent virulent and avirulent variants based on the previously performed *G. mellonella* virulence assay [35].

**Table 2:** The phenotypic and molecular characteristics of the examined *Pseudomonas aeruginosa* strains.

Biofilm-Forming Ability (48 h) [35]	Origin	*G. mellonella* Virulence (Survial %, 48 h) [35]	Multilocus Sequence Type (ST) [72,73]	Virulence Factors [35]							Biofilm-Forming Ability [33]	Antibiotic Resistance Phenotype [74]	Serotype [35]	Swimming [35]	Swarming [35]	Twitching [35]
				*exoS*	*exoU*	*lasB*	*algD*	*aprA*	*plcH*	Hemolysis						
ATCC 10145	Type strain, unknown source	n.d.	n.d.	n.d.	n.d.	n.d.	n.d.	n.d.	n.d.	n.d.	n.d.	n.d.	n.d.	n.d.	n.d.	n.d.
ATCC 15442	Water bottle in animal room	n.d.	252	n.d.	n.d.	n.d.	n.d.	n.d.	n.d.	n.d.	n.d.	n.d.	n.d.	n.d.	n.d.	n.d.
ATCC 27853	Clinical	15% (virulent)	155	+	–	+	+	+	+	+	++	Sensitive (10/0)	n.d.	n.d.	n.d.	n.d.
KPS-3	Clinical	35% (moderately virulent)	253	−	+	+	+	+	+	+	+++	Sensitive (10/0)	n.d.	n.d.	n.d.	n.d.
P9	Hydrocarbon-contaminated groundwater	75% (avirulent)	377	−	+	+	+	+	+	−	++	Resistant (10/1)	ONT	NM	NM	M
**P14**	**Hydrocarbon-contaminated soil**	**95% (avirulent)**	2586 *	**−**	**+**	**+**	**+**	**+**	**+**	**+/−**	**+++**	**Resistant (10/5)**	**O3**	**HM**	**NM**	**M**
P18	Hydrocarbon-contaminated soil	90% (avirulent)	3243 *	−	+	+	+	+	+	+/−	+	Sensitive (10/0)	O3	M	M	M
P43	Hydrocarbon-contaminated groundwater	90% (avirulent)	253	+	–	+	+	+	+	+	+	Multidrug-resistant (10/6)	ONT	M	M	M
**P66**	**Hydrocarbon-contaminated groundwater**	**5% (virulent)**	**455**	**+**	**–**	**+**	**+**	**+**	**+**	**+++**	**+++**	**Sensitive (10/0)**	**O11**	**HM**	**M**	**HM**
P69	Hydrocarbon-contaminated soil	50% (weakly virulent)	439	−	+	+	+	+	+	++	−	Resistant (10/5)	O6	M	M	M
P114	Compost	15% (virulent)	3255 *	+	–	+	+	+	+	+++	+	Resistant (10/4)	O1	HM	HM	HM
P135	Hydrocarbon-contaminated soil	70% (weakly virulent)	3257 *	−	–	+	+	+	+	+++	+	Intermediate (10/1)	O3	HM	M	HM
P144	Hydrocarbon-contaminated soil	0% (virulent)	1411	−	+	+	−	−	−	+	−	Sensitive (10/0)	O1	HM	M	HM
P164	Sewage	0% (virulent)	3260 *	+	−	+	+	+	−	++	−	Sensitive (10/0)	O11	M	NM	M
P177	Hydrocarbon-contaminated groundwater	30% (moderately virulent)	3262 *	+	−	+	+	+	+	++	+	Sensitive (10/0)	O6	HM	M	HM

Bold: strains used for preliminary screening; n.d., no data; * unique sequence type. Virulence factors: + positive PCR; – negative PCR. Hemolysis on blood agar: − no hemolysis; + moderate hemolysis, ++ normal hemolysis, +++ intensive hemolysis. Biofilm forming in a microtiter assay: – no biofilm producer; + weak biofilm producer; ++ moderate biofilm producer; +++ strong biofilm producer. Motility: HM—hypermotile; M—motile; NM—non-motile.

### 4.3. Preparation of Bacterial Suspensions

Bacterial cultivation was performed in Luria-Bertani (LB) broth (10.0 g tryptone, 5.0 g yeast extract and 9.0 g NaCl in 1000 mL distilled water), a medium that was proved to be non-toxic to zebrafish larvae in a previous experiment [75]. Overnight liquid cultures of the examined *P. aeruginosa* strains were diluted with fresh LB to reach an optical density of OD600 = 0.6 ± 0.02 (stock solution) and were serially diluted to reach the final infectious dose.

### 4.4. Zebrafish Maintenance and Egg Collection

The zebrafish were kept and bred according to the general protocol of the MATE zebrafish lab, which was described in the article by Csenki et al. [30]. In brief: AB strain zebrafish were held in breeding groups (30 females and 30 males) in a Tecniplast ZebTEC recirculation system (Tecniplast S.p.A., Buguggiate, Italy) at 25.5 °C ± 0.5 °C, pH 7.0 ± 0.2, conductivity 550 ± 50 mS/cm (system water) and light:dark period of 14 h:10 h. Fish were fed twice a day with granulate fishfood (Zebrafeed 400–600 mm, Sparos Lda., Olhão, Portugal) supplemented with freshly hatched live Artemia salina twice a week. Fish were placed in breeding tanks (Tecniplast S.p.a., Buguggiate, Italy) late in the afternoon the day before the microinjection experiments. Spawning of individual pairs was delayed through time to allow a continuous supply of 1-cell embryos.

### 4.5. Microinjection

Microinjection was conducted as described by Csenki et al. [30] using the above-described bacterial suspensions in LB. The effect of LB medium on the injected embryos in the largest injectable droplet size (200 μm) was previously tested and proved to be safe for application [30,75,76]. The microinjections were carried out directly into the yolk (Y) or into the perivitelline space (PV) using three different sphere diameters (50 μm, 75 μm and 200 μm) corresponding to an injection volume of 0.074 nL, 0.22 nL and 4.17 nL, respectively, using the tenfold dilution series of the stock bacterial suspension (10^1^–10^4^) (the embryos received only one type of treatment). Determination of injection volumes was previously described, and the method was validated to be dimensionally stable [30]. Treatment was performed in five replicates with ten embryos in each experimental group (n = 50).

### 4.6. Endpoints of the Virulence Test/Examination of Injected Embryos

Two hours after injection, coagulated and/or non-fertilized eggs were discarded, and developing embryos were transferred in groups of ten into 6 cm diameter Petri dishes filled with sterilized E3 medium (5 mM NaCl, 0.17 mM KCl, 0.4 mM CaCl_2_ and 0.16 mM MgSO_4_). Microinjected embryos were incubated at 25 ± 1 °C in Memmert Thermostat to ensure the optimal temperature for both test organisms. Treatments were performed in five replicates in the case of optimization experiments and in three in the case of validation studies. Embryo mortality and sublethal symptoms were monitored daily. Embryo mortality was determined on the basis of egg coagulation, the lack of somite formation and the lack of heart function. Pericardial edema, yolk edema, tail deformity, craniofacial deformity and disintegrated abnormal embryo shape were defined as sublethal endpoints [77]. Digital images were captured of laterally oriented larvae at 30× magnification using a stereomicroscope (Leica M205 FA, Leica DFC 7000T camera, Leica Application Suite X, Leica Microsystems GmbH, Wetzlar, Germany).

### 4.7. Statistical Analysis

Normality of the data was analyzed in the R software version 4.2.1. [78] with Shapiro–Wilk normality test [79], and homogeneity of variances was tested with Bartlett test [80]. Statistical data analysis of the exposition routes, drop sizes, bacterial concentrations and incubation times and visualization were performed using GraphPad Prism 9 software, version 9.5.1. (GraphPad Software Inc., San Diego, CA, USA), and R software, version 4.2.1. [78], with ordinary one-way ANOVA followed by Dunnet’s multiple-comparison test with a confidence interval of 95%.

## 5. Conclusions

Methods based on zebrafish embryo microinjection play a significant role in testing the infectivity of various microorganisms, which are primarily used to qualify nosocomial isolates. These studies take advantage of the immunological similarities between zebrafish embryos and higher vertebrates and reflect the mechanisms of infections quite well. However, these methods are often difficult to implement and only give an idea of direct infection.

The two types of microinjection procedures presented in this study are able to simultaneously qualify the real infectivity of the examined microbial strain, as well as the effects that develop after early direct infection. As can be seen from our results, significant differences are detectable between the different types of expositions, even in the case of the same strain. The major advantage of in vivo procedures like microinjection is that they have a low budget, do not require special equipment and are easy to learn. By following the optimized steps, our newly developed method can be easily adapted to other microbial species, regardless of their fast-growing or slow-growing characteristics or their phenotypic traits such as motility. After adapting the method to the microbial species to be tested, the method enables the qualification of a given microbial strain in up to 3 days, for which it is sufficient to use only mortality as the endpoint. The newly developed method is primarily recommended for the qualification of environmental isolates.

## Figures and Tables

**Table 1:** Settings used for the optimization of the zebrafish microinjection virulence model.

		Levels of Dilution and Bacterial Density (CFU) of the Final Injection Volume (SD = ±3.33%)			
Exposition	Drop Size	10^−1^	10^−2^	10^−3^	10^−4^
Perivitelline space (PV)	100 µM (0.52 nL)	2.4 × 10^1^	2.4 × 10^0^	2.4 × 10^−1^	
	150 µM (1.77 nL)	8.4 × 10^1^	8.4 × 10^0^ *	8.4 × 10^−1^	
	200 µM (4.17 nL)	2.0 × 10^2^	2.0 × 10^1^	2.0 × 10^0^	
Yolk (Y)	100 µM (0.52 nL)		2.4 × 10^0^	2.4 × 10^−1^	2.4 × 10^−2^
	150 µM (1.77 nL)		8.4 × 10^0^ *	8.4 × 10^−1^	8.4 × 10^−2^
	200 µM (4.17 nL)		2.0 × 10^1^	2.0 × 10^0^	2.0 × 10^−1^

* Minimum infectious dose (MID); red color—bacterial densities above MID; blue color—bacterial densities below MID.

## Data Availability

Data are available upon request by email to the corresponding author.

## Supplementary Materials

The following supporting information can be downloaded at https://www.mdpi.com/article/10.3390/antibiotics12121740/s1. Table S1: The comparison of mortality results of the examined *P. aeruginosa* obtained by a previous *G. mellonella* test and the newly developed zebrafish microinjection method

## References

[1] Hardalo C., Edberg S.C. (2008). Pseudomonas Aeruginosa: Assessment of Risk from Drinking Water. Crit. Rev. Microbiol..

[2] Aliaga L., Mediavilla J.D., Cobo F. (2002). A Clinical Index Predicting Mortality with Pseudomonas Aeruginosa Bacteraemia. J. Med. Microbiol..

[3] Thaden J.T., Park L.P., Maskarinec S.A., Ruffin F., Fowler V.G., Van Duin D. (2017). Results from a 13-Year Prospective Cohort Study Show Increased Mortality Associated with Bloodstream Infections Caused by Pseudomonas Aeruginosa Compared to Other Bacteria. Antimicrob. Agents Chemother..

[4] Sood U., Hira P., Kumar R., Bajaj A., Rao D.L.N., Lal R., Shakarad M. (2019). Comparative Genomic Analyses Reveal Core-Genome-Wide Genes under Positive Selection and Major Regulatory Hubs in Outlier Strains of Pseudomonas Aeruginosa. Front. Microbiol..

[5] Cabot G., Zamorano L., Moyà B., Juan C., Navas A., Blázquez J., Oliver A. (2016). Evolution of Pseudomonas Aeruginosa Antimicrobial Resistance and Fitness under Low and High Mutation Rates. Antimicrob. Agents Chemother..

[6] Stover C.K., Pham X.Q., Erwin A.L., Mizoguchi S.D., Warrener P., Hickey M.J., Brinkman F.S.L., Hufnagle W.O., Kowallk D.J., Lagrou M. (2000). Complete Genome Sequence of Pseudomonas Aeruginosa PAO1, an Opportunistic Pathogen. Nature.

[7] Chaerun S.K., Tazaki K., Asada R., Kogure K. (2004). Bioremediation of Coastal Areas 5 Years after the Nakhodka Oil Spill in the Sea of Japan: Isolation and Characterization of Hydrocarbon-Degrading Bacteria. Environ. Int..

[8] Huang H., Shao X., Xie Y., Wang T., Zhang Y., Wang X., Deng X. (2019). An Integrated Genomic Regulatory Network of Virulence-Related Transcriptional Factors in Pseudomonas Aeruginosa. Nat. Commun..

[9] Liao C., Huang X., Wang Q., Yao D., Lu W. (2022). Virulence Factors of Pseudomonas Aeruginosa and Antivirulence Strategies to Combat Its Drug Resistance. Front. Cell Infect. Microbiol..

[10] Strateva T., Mitov I. (2011). Contribution of an Arsenal of Virulence Factors to Pathogenesis of Pseudomonas Aeruginosa Infections. Ann. Microbiol..

[11] Algammal A.M., Mabrok M., Sivaramasamy E., Youssef F.M., Atwa M.H., El-kholy A.W., Hetta H.F., Hozzein W.N. (2020). Emerging MDR-Pseudomonas Aeruginosa in Fish Commonly Harbor OprL and ToxA Virulence Genes and BlaTEM, BlaCTX-M, and TetA Antibiotic-Resistance Genes. Sci. Rep..

[12] Wolfgang M.C., Kulasekara B.R., Liang X., Boyd D., Wu K., Yang Q., Miyada C.G., Lory S. (2003). Conservation of Genome Content and Virulence Determinants among Clinical and Environmental Isolates of Pseudomonas Aeruginosa. Proc. Natl. Acad. Sci. USA.

[13] Poulsen B.E., Yang R., Clatworthy A.E., White T., Osmulski S.J., Li L., Penaranda C., Lander E.S., Shoresh N., Hung D.T. (2019). Defining the Core Essential Genome of Pseudomonas Aeruginosa. Proc. Natl. Acad. Sci. USA.

[14] Freschi L., Vincent A.T., Jeukens J., Emond-Rheault J.G., Kukavica-Ibrulj I., Dupont M.J., Charette S.J., Boyle B., Levesque R.C. (2019). The Pseudomonas Aeruginosa Pan-Genome Provides New Insights on Its Population Structure, Horizontal Gene Transfer, and Pathogenicity. Genome Biol. Evol..

[15] Wei L., Wu Q., Zhang J., Guo W., Gu Q., Wu H., Wang J., Lei T., Xue L., Zhang Y. (2020). Prevalence, Virulence, Antimicrobial Resistance, and Molecular Characterization of Pseudomonas Aeruginosa Isolates From Drinking Water in China. Front. Microbiol..

[16] Castañeda-Montes F.J., Avitia M., Sepúlveda-Robles O., Cruz-Sánchez V., Kameyama L., Guarneros G., Escalante A.E. (2018). Population Structure of Pseudomonas Aeruginosa through a MLST Approach and Antibiotic Resistance Profiling of a Mexican Clinical Collection. Infect. Genet. Evol..

[17] Ali N.G., Ali T.E.S., Aboyadak I.M., Elbakry M.A. (2021). Controlling Pseudomonas Aeruginosa Infection in Oreochromis Niloticus Spawners by Cefotaxime Sodium. Aquaculture.

[18] Jose D., Mohandas A., Singh I.S.B. (2018). A Non-Pathogenic Environmental Isolate of Pseudomonas Aeruginosa MCCB 123 with Biotechnological Potential. Int.J.Curr.Microbiol.App.Sci..

[19] Radhapriya P., Ramachandran A., Anandham R., Mahalingam S. (2015). Pseudomonas Aeruginosa RRALC3 Enhances the Biomass, Nutrient and Carbon Contents of Pongamia Pinnata Seedlings in Degraded Forest Soil. PLoS ONE.

[20] Berger C., Rückert C., Blom J., Rabaey K., Kalinowski J., Rosenbaum M.A. (2021). Estimation of Pathogenic Potential of an Environmental Pseudomonas Aeruginosa Isolate Using Comparative Genomics. Sci. Rep..

[21] Ebadi A., Khoshkholgh Sima N.A., Olamaee M., Hashemi M., Ghorbani Nasrabadi R. (2017). Effective Bioremediation of a Petroleum-Polluted Saline Soil by a Surfactant-Producing Pseudomonas Aeruginosa Consortium. J. Adv. Res..

[22] Yasmin S., Hafeez F.Y., Mirza M.S., Rasul M., Arshad H.M.I., Zubair M., Iqbal M. (2017). Biocontrol of Bacterial Leaf Blight of Rice and Profiling of Secondary Metabolites Produced by Rhizospheric Pseudomonas Aeruginosa BRp3. Front. Microbiol..

[23] Llamas M.A., van der Sar A.M. (2014). Assessing Pseudomonas Virulence with Nonmammalian Host: Zebrafish. Methods Mol. Biol..

[24] Lorenz A., Pawar V., Häussler S., Weiss S. (2016). Insights into Host–Pathogen Interactions from State-of-the-Art Animal Models of Respiratory Pseudomonas Aeruginosa Infections. FEBS Lett..

[25] Pont S., Blanc-Potard A.B. (2021). Zebrafish Embryo Infection Model to Investigate Pseudomonas Aeruginosa Interaction With Innate Immunity and Validate New Therapeutics. Front. Cell Infect. Microbiol..

[26] Meijer A.H., Spaink H.P. (2011). Host-Pathogen Interactions Made Transparent with the Zebrafish Model. Curr. Drug Targets.

[27] Gomes M.C., Mostowy S. (2020). The Case for Modeling Human Infection in Zebrafish. Trends Microbiol..

[28] Benard E.L., van der Sar A.M., Ellett F., Lieschke G.J., Spaink H.P., Meijer A.H. (2012). Infection of Zebrafish Embryos with Intracellular Bacterial Pathogens. J. Vis. Exp..

[29] Carvalho R., de Sonneville J., Stockhammer O.W., Savage N.D.L., Veneman W.J., Ottenhoff T.H.M., Dirks R.P., Meijer A.H., Spaink H.P. (2011). A High-Throughput Screen for Tuberculosis Progression. PLoS ONE.

[30] Csenki Z., Garai E., Risa A., Cserháti M., Bakos K., Márton D., Bokor Z., Kriszt B., Urbányi B. (2019). Biological Evaluation of Microbial Toxin Degradation by Microinjected Zebrafish (*Danio Rerio*) Embryos. Chemosphere.

[31] Nogaret P., El Garah F., Blanc-Potard A.B. (2021). A Novel Infection Protocol in Zebrafish Embryo to Assess Pseudomonas Aeruginosa Virulence and Validate Efficacy of a Quorum Sensing Inhibitor in Vivo. Pathogens.

[32] Rowe H.M., Withey J.H., Neely M.N. (2014). Zebrafish as a Model for Zoonotic Aquatic Pathogens. Dev. Comp. Immunol..

[33] Díaz-Pascual F., Ortíz-Severín J., Varas M.A., Allende M.L., Chávez F.P. (2017). In Vivo Host-Pathogen Interaction as Revealed by Global Proteomic Profiling of Zebrafish Larvae. Front. Cell Infect. Microbiol..

[34] Poplimont H., Georgantzoglou A., Boulch M., Walker H.A., Coombs C., Papaleonidopoulou F., Sarris M. (2020). Neutrophil Swarming in Damaged Tissue Is Orchestrated by Connexins and Cooperative Calcium Alarm Signals. Curr. Biol..

[35] Kaszab E., Radó J., Kriszt B., Pászti J., Lesinszki V., Szabó Á., Tóth G., Khaledi A., Szoboszlay S. (2019). Groundwater, Soil and Compost, as Possible Sources of Virulent and Antibiotic-Resistant Pseudomonas Aeruginosa. Int. J. Environ. Health Res..

[36] Lizewski S.E., Lundberg D.S., Schurr M.J. (2002). The Transcriptional Regulator AlgR Is Essential for Pseudomonas Aeruginosa Pathogenesis. Infect. Immun..

[37] Roser D.J., Van Den Akker B., Boase S., Haas C.N., Ashbolt N.J., Rice S.A. (2015). Dose–Response Algorithms for Water-Borne Pseudomonas Aeruginosa Folliculitis. Epidemiol. Infect..

[38] Deredjian A., Colinon C., Hien E., Brothier E., Youenou B., Cournoyer B., Dequiedt S., Hartmann A., Jolivet C., Houot S. (2014). Low Occurrence of Pseudomonas Aeruginosa in Agricultural Soils with and without Organic Amendment. Front. Cell Infect. Microbiol..

[39] Kaszab E., Szoboszlay S., Dobolyi C., Háhn J., Pék N., Kriszt B. (2011). Antibiotic Resistance Profiles and Virulence Markers of Pseudomonas Aeruginosa Strains Isolated from Composts. Bioresour. Technol..

[40] Mena K.D., Gerba C.P. (2009). Risk Assessment of Pseudomonas Aeruginosa in Water. Rev. Env. Contam. Toxicol..

[41] Kaszab E., Kriszt B., Atzél B., Szabó G., Szabó I., Harkai P., Szoboszlay S. (2010). The Occurrence of Multidrug-Resistant Pseudomonas Aeruginosa on Hydrocarbon-Contaminated Sites. Microb. Ecol..

[42] Chen Z.Y., Li N.J., Cheng F.Y., Hsueh J.F., Huang C.C., Lu F.I., Fu T.F., Yan S.J., Lee Y.H., Wang Y.J. (2020). The Effect of the Chorion on Size-Dependent Acute Toxicity and Underlying Mechanisms of Amine-Modified Silver Nanoparticles in Zebrafish Embryos. Int. J. Mol. Sci..

[43] Diggle S.P., Whiteley M. (2020). Microbe Profile: Pseudomonas Aeruginosa: Opportunistic Pathogen and Lab Rat. Microbiology.

[44] Rosen J.N., Sweeney M.F., Mably J.D. (2009). Microinjection of Zebrafish Embryos to Analyze Gene Function. J. Vis. Exp..

[45] Walker M.K., Hufnagle L.C., Clayton M.K., Peterson R.E. (1992). An Egg Injection Method for Assessing Early Life Stage Mortality of Polychlorinated Dibenzo-p-Dioxins, Dibenzofurans, and Biphenyls in Rainbow Trout, (*Oncorhynchus Mykiss*). Aquat. Toxicol..

[46] Schubert S., Keddig N., Hanel R., Kammann U. (2014). Microinjection into Zebrafish Embryos (*Danio Rerio*)—A Useful Tool in Aquatic Toxicity Testing?. Environ. Sci. Eur..

[47] Xie J., He J.B., Shi J.W., Xiao Q., Li L., Woo P.C.Y. (2014). An Adult Zebrafish Model for Laribacter Hongkongensis Infection: Koch’s Postulates Fulfilled. Emerg. Microbes Infect..

[48] Bose S., Ghosh A.K. (2015). Diagnosis of Biofilm-Associated Infections in Medical Devices. Biomater. Med. Device-Assoc. Infect..

[49] Velikova N., Kavanagh K., Wells J.M. (2016). Evaluation of Galleria Mellonella Larvae for Studying the Virulence of Streptococcus Suis. BMC Microbiol..

[50] Clatworthy A.E., Lee J.S.W., Leibman M., Kostun Z., Davidson A.J., Hung D.T. (2009). Pseudomonas Aeruginosa Infection of Zebrafish Involves Both Host and Pathogen Determinants. Infect. Immun..

[51] Llamas M.A., Van Der Sar A., Chu B.C.H., Sparrius M., Vogel H.J., Bitter W. (2009). A Novel Extracytoplasmic Function (ECF) Sigma Factor Regulates Virulence in Pseudomonas Aeruginosa. PLoS Pathog..

[52] Wood S.J., Kuzel T.M., Shafikhani S.H. (2023). Pseudomonas Aeruginosa: Infections, Animal Modeling, and Therapeutics. Cells.

[53] Kukavica-Ibrulj I., Bragonzi A., Paroni M., Winstanley C., Sanschagrin F., O’Toole G.A., Levesque R.C. (2008). In Vivo Growth of Pseudomonas Aeruginosa Strains PAO1 and PA14 and the Hypervirulent Strain LESB58 in a Rat Model of Chronic Lung Infection. J. Bacteriol..

[54] Van Heeckeren A.M., Schluchter M.D. (2002). Murine Models of Chronic Pseudomonas Aeruginosa Lung Infection. Lab. Anim..

[55] Kaszab E., Szoboszlay S., Dura G., Radó J., Kovács B., Kriszt B. (2016). Pathogenic and Phylogenetic Features of 2 Multiresistant Pseudomonas Aeruginosa Strains Originated from Remediated Sites. Int. J. Occup. Med. Environ. Health.

[56] Fauvarque M.O., Bergeret E., Chabert J., Dacheux D., Satre M., Attree I. (2002). Role and Activation of Type III Secretion System Genes in Pseudomonas Aeruginosa-Induced Drosophila Killing. Microb. Pathog..

[57] Tan M.W., Rahme L.G., Sternberg J.A., Tompkins R.G., Ausubel F.M. (1999). Pseudomonas Aeruginosa Killing of Caenorhabditis Elegans Used to Identify P. Aeruginosa Virulence Factors. Proc. Natl. Acad. Sci. USA.

[58] Tan M.W., Mahajan-Miklos S., Ausubel F.M. (1999). Killing of Caenorhabditis Elegans by Pseudomonas Aeruginosa Used to Model Mammalian Bacterial Pathogenesis. Proc. Natl. Acad. Sci. USA.

[59] Miyata S., Casey M., Frank D.W., Ausubel F.M., Drenkard E. (2003). Use of the Galleria Mellonella Caterpillar as a Model Host to Study the Role of the Type III Secretion System in Pseudomonas Aeruginosa Pathogenesis. Infect. Immun..

[60] Kumar S.S., Tandberg J.I., Penesyan A., Elbourne L.D.H., Suarez-Bosche N., Don E., Skadberg E., Fenaroli F., Cole N., Winther-Larsen H.C. (2018). Dual Transcriptomics of Host-Pathogen Interaction of Cystic Fibrosis Isolate Pseudomonas Aeruginosa PASS1 With Zebrafish. Front. Cell Infect. Microbiol..

[61] Rocker A.J., Weiss A.R.E., Lam J.S., Van Raay T.J., Khursigara C.M. (2015). Visualizing and Quantifying Pseudomonas Aeruginosa Infection in the Hindbrain Ventricle of Zebrafish Using Confocal Laser Scanning Microscopy. J. Microbiol. Methods.

[62] Kiani A.K., Pheby D., Henehan G., Brown R., Sieving P., Sykora P., Marks R., Falsini B., Capodicasa N., Miertus S. (2022). Ethical Considerations Regarding Animal Experimentation. J. Prev. Med. Hyg..

[63] Directive 2010/63/EU of the European Parliament and of the Council of 22 September 2010 on the Protection of Animals Used for Scientific Purposes (Text with EEA Relevance). https://eur-lex.europa.eu/LexUriServ/LexUriServ.do?uri=OJ:L:2010:276:0033:0079:en:PDF.

[64] (2020). Commission Implementing Decision (EU) 2020/569 of 16 April 2020 Establishing a Common Format and Information Content for the Submission of the Information to Be Reported by Member States Pursuant to Directive 2010/63/EU of the European Parliament and of the Council on the Protection of Animals Used for Scientific Purposes and Repealing Commission Implementing Decision 2012/707/EU (Notified under Document C(2020) 2179) (Text with EEA Relevance), C/2020/2179. https://eur-lex.europa.eu/legal-content/EN/TXT/?uri=CELEX%3A32020D0569.

[65] Bondue T., Berlingerio S.P., van den Heuvel L., Levtchenko E. (2023). The Zebrafish Embryo as a Model Organism for Testing MRNA-Based Therapeutics. Int. J. Mol. Sci..

[66] Zhang X., Zhao Y., Wu Q., Lin J., Fang R., Bi W., Dong G., Li J., Zhang Y., Cao J. (2019). Zebrafish and Galleria Mellonella: Models to Identify the Subsequent Infection and Evaluate the Immunological Differences in Different Klebsiella Pneumoniae Intestinal Colonization Strains. Front. Microbiol..

[67] Liew S.M., Rajasekaram G., Puthucheary S.D.A., Chua K.H. (2019). Antimicrobial Susceptibility and Virulence Genes of Clinical and Environmental Isolates of Pseudomonas Aeruginosa. PeerJ.

[68] Woods D.E., Lam J.S., Paranchych W., Speert D.P., Campbell M., Godfrey A.J. (2011). Correlation of Pseudomonas Aeruginosa Virulence Factors from Clinical and Environmental Isolates with Pathogenicity in the Neutropenic Mouse. Can. J. Microbiol..

[69] Vives-Flórez M., Garnica D. (2006). Comparison of Virulence between Clinical and Environmental *Pseudomonas Aeruginosa* Isolates. Int. Microbiol..

[70] Wang Y., Li C., Gao C., Ma C., Xua P. (2014). Genome Sequence of the Nonpathogenic Pseudomonas Aeruginosa Strain ATCC 15442. Genome Announc..

[71] Atzél B., Szoboszlay S., Mikuska Z., Kriszt B. (2008). Comparison of Phenotypic and Genotypic Methods for the Detection of Environmental Isolates of Pseudomonas Aeruginosa. Int. J. Hyg. Environ. Health.

[72] PubMLST—Public Databases for Molecular Typing and Microbial Genome Diversity. https://pubmlst.org/.

[73] Radó J., Kaszab E., Petrovics T., Pászti J., Kriszt B., Szoboszlay S. (2017). Characterization of Environmental Pseudomonas Aeruginosa Using Multilocus Sequence Typing Scheme. J. Med. Microbiol..

[74] EUCAST: Clinical Breakpoints and Dosing of Antibiotics. https://www.eucast.org/clinical_breakpoints/.

[75] Garai E., Risa A., Varga E., Cserháti M., Kriszt B., Urbányi B., Csenki Z. (2021). Evaluation of the Multimycotoxin-Degrading Efficiency of Rhodococcus Erythropolis NI1 Strain with the Three-Step Zebrafish Microinjection Method. Int. J. Mol. Sci..

[76] Garai E., Risa A., Varga E., Cserháti M., Kriszt B., Urbányi B., Csenki Z. (2020). Qualifying the T-2 Toxin-Degrading Properties of Seven Microbes with Zebrafish Embryo Microinjection Method. Toxins.

[77] Csenki Z., Bartók T., Bock I., Horváth L., Lemli B., Zsidó B.Z., Angeli C., Hetényi C., Szabó I., Urbányi B. (2023). Interaction of Fumonisin B1, N-Palmitoyl-Fumonisin B1, 5-O-Palmitoyl-Fumonisin B1, and Fumonisin B4 Mycotoxins with Human Serum Albumin and Their Toxic Impacts on Zebrafish Embryos. Biomolecules.

[78] R: The R Project for Statistical Computing. https://www.r-project.org/.

[79] Royston J.P. (1982). An Extension of Shapiro and Wilk’s W Test for Normality to Large Samples. Appl. Stat..

[80] (1937). Properties of Sufficiency and Statistical Tests. Proc. R. Soc. Lond. A Math. Phys. Sci..

