# Supplementary material for: Evaluating the In Vivo Virulence of Environmental Pseudomonas aeruginosa Using Microinjection Model of Zebrafish (Danio rerio)
Source: Antibiotics (Basel). 2023 Dec 15;12(12):1740. doi: 10.3390/antibiotics12121740 (PMC10740789; doi:10.3390/antibiotics12121740)
Supplement: Supplementary file 1 [file antibiotics-12-01740-s001.zip › antibiotics-2761081-supplementary.pdf]

Supplementary Table S1. The comparison of mortality results of the examined *P. aeruginosa* obtained by a previous *G. mellonella* test and the newly developed zebrafish microinjection method.

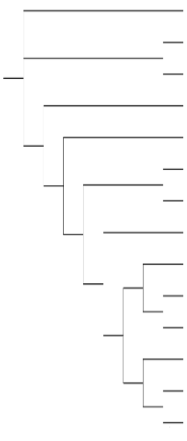

**Multilocus sequence type (ST)** [72,73]	**Designation of *P. aeruginosa* strain**	***G. mellonella* test (survival%, 48h)** [35]	**Zebrafish microinjection (average survival% of 3 repeated tests and**		**Virulence factors** [35]							**Biofilm forming ability (48h)** [35]	**Antibiotic resistance phenotype** [35,39,41,74]
			**Y**	**PV**	***exoS***	***exoU***	***lasB***	***algD***	***aprA***	***plcH***	**hemolysis**		
253	KPS–3	35%	54%	92%	–	+	+	+	+	+	+	+++	Sensitive (10/0)
253	P43	90%	34%	82%	+	–	+	+	+	+	+	+	Multidrug resistant (10/6)
252	ATCC 15442	n.d.	98%	90%	n.d.	n.d.	n.d.	n.d.	n.d.	n.d.	n.d.	n.d.	n.d.
377	P9	75%	76%	74%	–	+	+	+	+	+	–	++	Resistant (10/1)
1411	P144	0%	78%	76%	–	+	+	–	–	–	+	–	Sensitive (10/0)
3260*	P164	0%	76%	78%	+	–	+	+	+	–	++	–	Sensitive (10/0)
3257*	P135	70%	36%	80%	–	–	+	+	+	+	+++	+	Intermediate (10/1)
**455**	**P66**	**5%**	**17%**	**56%**	**+**	**–**	**+**	**+**	**+**	**+**	**+++**	**+++**	**Sensitive (10/0)**
439	P69	50%	20%	60%	–	+	+	+	+	+	++	–	Resistant (10/5)
3243*	P18	90%	0%	53%	–	+	+	+	+	+	+/–	+	Sensitive (10/0)
**2586***	**P14**	**95%**	**3 %**	**46%**	**–**	**+**	**+**	**+**	**+**	**+**	**+/–**	**+++**	**Resistant (10/5)**
3262*	P177	30%	76%	88%	+	–	+	+	+	+	++	+	Sensitive (10/0)
3255*	P114	15%	66%	94%	+	–	+	+	+	+	+++	+	Resistant (10/4)
155	ATCC 27853	15%	66%	98%	+	–	+	+	+	+	+	++	Sensitive (10/0)
n.d.	ATCC10145	n.d.	86%	80%	n.d.	n.d.	n.d.	n.d.	n.d.	n.d.	n.d.	n.d.	n.d.

Phylogenetic tree was generated by the multilocus sequence types (STs) of the examined strains using PUBMLST, the public database for molecular typing and microbial genome diversity (ATCC10145 was not classified). Bold: strains used for preliminary screening; n.d., no data; ; * unique sequence type; Y – yolk injection; PV – perivitellinar injection; avirulent: survival rate of 75─100%; weakly–virulent: survival rate of 50─74%; moderately virulent: survival rate of 25─49%); virulent: survival rate: 0─24%; Virulence factors: + positive PCR; – negative PCR; Hemolysis on blood agar: – no hemolysis; + moderate hemolysis, ++ normal hemolysis, +++ intensive hemolysis; Biofilm–forming in a microtiter assay: – no biofilm producer; + weak biofilm producer; ++ moderate biofilm producer; +++ strong biofilm producer.
